# Preoperative troponin and the interpretation of perioperative myocardial injury prediction

**DOI:** 10.1093/ehjdh/ztag150

**Published:** 2026-09-18

**Authors:** Güney Sarıoğlu

**Affiliations:** Department of Cardiology, Ministry of Health, Battalgazi State Hospital, Malatya 44320, Turkey

Sailer *et al*.^1^ present an important and clinically relevant effort to improve perioperative myocardial injury (PMI) risk prediction using an interpretable machine-learning model based on routinely available preoperative data. Their temporally validated Explainable Boosting Machine achieved better discrimination than conventional approaches and may offer a practical route towards more efficient postoperative troponin surveillance.

One aspect of the outcome definition, however, may be important for interpreting what the model is actually predicting. Perioperative myocardial injury was defined as postoperative high-sensitivity cardiac troponin above the upper reference limit.^1^ Yet preoperative troponin values were available in the cohort, and patients classified as having PMI had higher baseline troponin concentrations than those without PMI. This raises the possibility that some patients designated as having ‘perioperative myocardial injury’ already had elevated troponin before surgery.

That distinction is clinically meaningful. Contemporary perioperative frameworks emphasize the value of a preoperative troponin baseline when interpreting postoperative elevations, particularly because chronic myocardial injury is common in older patients and in those with renal dysfunction, heart failure, or other cardiovascular disease.^2–4^ In patients with an already elevated baseline value, a postoperative concentration above the assay upper reference limit does not necessarily indicate newly occurring perioperative myocardial injury. Consequently, a model trained on postoperative positivity alone may partly identify patients with persistent chronic troponin elevation rather than patients who develop incident perioperative injury.

This issue also affects interpretation of the model’s leading predictors. Age and renal function were among the strongest contributors to prediction,^1^ both of which are closely associated with chronically elevated high-sensitivity troponin concentrations. Their predictive importance may therefore reflect, at least in part, baseline myocardial injury rather than susceptibility to a new perioperative event.

I would therefore be interested to know what proportion of patients meeting the study’s PMI definition already had elevated preoperative troponin. A focused sensitivity analysis excluding such patients, or alternatively defining perioperative injury using a pre- to postoperative troponin change where baseline values were available, could clarify whether the model predicts incident perioperative injury or postoperative troponin elevation more broadly.

This distinction does not detract from the model’s predictive performance. Rather, it may help define its intended clinical use more precisely and determine whether it should be interpreted primarily as a tool for PMI prediction or for identifying patients likely to demonstrate postoperative troponin elevation.
